# A2AR Transmembrane 2 Peptide Administration Disrupts the A2AR-A2AR Homoreceptor but Not the A2AR-D2R Heteroreceptor Complex: Lack of Actions on Rodent Cocaine Self-Administration

**DOI:** 10.3390/ijms20236100

**Published:** 2019-12-03

**Authors:** Dasiel O. Borroto-Escuela, Karolina Wydra, Wilber Romero-Fernandez, Zilong Zhou, Malgorzata Frankowska, Malgorzata Filip, Kjell Fuxe

**Affiliations:** 1 Department of Neuroscience, Karolinska Institutet, 16175 Stockholm, Sweden; wromfdez@gmail.com (W.R.-F.); zilongzhou@outlook.com (Z.Z.); 2 Department of Biomolecular Science, Section of Physiology, University of Urbino, Campus Scientifico Enrico Mattei, via Ca’ le Suore 2, I-61029 Urbino, Italy; 3 Grupo Bohío-Estudio, Observatorio Cubano de Neurociencias, Zayas 50, Yaguajay 62100, Cuba; 4 Department of Drug Addiction Pharmacology, Maj Institute of Pharmacology Polish Academy of Sciences, 31-343 Kraków, Poland; wydra@if-pan.krakow.pl (K.W.); mal.fil@if-pan.krakow.pl (M.F.); frankow@if-pan.krakow.pl (M.F.); 5 Science for Life Laboratory, Department of Cell and Molecular Biology, Uppsala University, 752 36 Uppsala, Sweden; 6 National Engineering Laboratory for Druggable Gene and Protein Screening, Northeast Normal University, Changchun 130024, China

**Keywords:** adenosine A2A receptor, dopamine D2 receptor, homoreceptor complexes, heteroreceptor complexes, allosteric receptor–receptor interactions, cocaine use disorder, small interfering peptides: G protein-coupled receptors

## Abstract

It was previously demonstrated that rat adenosine A2AR transmembrane V peptide administration into the nucleus accumbens enhances cocaine self-administration through disruption of the A2AR-dopamine (D2R) heteroreceptor complex of this region. Unlike human A2AR transmembrane 4 (TM4) and 5 (TM5), A2AR TM2 did not interfere with the formation of the A2AR-D2R heteroreceptor complex in cellular models using BRET^1^ assay. A2AR TM2 was proposed to be part of the of the receptor interface of the A2AR homomer instead and was therefore tested in the current article for effects on rat cocaine self-administration using rat A2AR synthetic TM2 peptide bilaterally injected into the nucleus accumbens. The injected A2AR TM2 peptide failed to significantly counteract the inhibitory action of the A2AR agonist CGS 21680 (0.1 mg/Kg) on cocaine self-administration. In line with these results, the microinjected A2AR TM2 peptide did not reduce the number of proximity ligation assay blobs identifying A2AR-D2R heteroreceptor complexes in the nucleus accumbens. In contrast, the A2AR TM2 peptide significantly reduced the number of A2AR-A2AR homoreceptor complexes in the nucleus accumbens. As to effects on the receptor–receptor interactions in the A2AR-D2R heteroreceptor complexes, the A2AR TM2 peptide did not alter the significant increase in the D2R Ki, high values produced by the A2AR agonist CGS 21680 ex vivo in the ventral striatum. The results indicate that the accumbal A2AR-A2AR homomeric complexes are not involved in mediating the A2AR agonist-induced inhibition of cocaine self-administration.

## 1. Introduction

The concept was introduced in 1993 that allosteric receptor–receptor interactions in the plasma membrane can develop due to their existence in receptor heterodimers [1]. The antagonistic receptor–receptor interactions in the A2AR-D2R heteromer was of special interest in view of its inhibition of D2R protomer recognition and signaling [2]. This was in line with the ability of adenosine receptor antagonists to enhance the antiparkinsonian actions of DA receptor agonists in the rat hemiparkinson model [3]. Antagonistic A2AR-D2R interactions may play a relevant role in Parkinson’s disease, schizophrenia, and cocaine addiction [4,5,6,7,8,9].

A molecular resolution model of the A2AR-D2R heteromer was established [10]. The human A2AR TM5 was demonstrated to be part of its receptor interface [10,11,12]. Microinjection of the rat A2AR TM5 into the nucleus accumbens was found to disrupt the A2AR-D2R heteroreceptor complexes in this region and to enhance cocaine self-administration [12]. In contrast, the human A2AR-TM2 (KKK-FFVVSLAAADIAVGVLAIPFAITI-KKK) peptide was not found to be part of the A2AR-D2R interface [10,11]. Instead, there are indications that the rat A2AR synthetic TM2 peptide may be part of the receptor interface of the A2AR-A2AR homoreceptor complex based on BRET^1^ experiments [10].

In the current experiments, the rat synthetic A2AR TM2 peptide was therefore microinjected into the nucleus accumbens to test if it could block the inhibitory effects of A2AR agonist CGS 21680 on cocaine self-administration. Furthermore, by means of in situ proximity ligation assay (in situ PLA), it was studied if the A2AR-A2AR homoreceptor complexes and A2AR-D2R heteroreceptor complexes were disrupted by the TM2 small synthetic peptides microinjections. Possible changes induced by the rat A2AR TM2 peptide in the antagonistic allosteric receptor–receptor interactions of the A2AR-D2R heteroreceptor complexes were evaluated by using radioligand binding assays. The A2AR agonist CGS 21680 was also added ex vivo to activate the antagonistic A2AR-D2R interaction.

## 2. Results

### 2.1. Effects of Intra-Accumbal Microinjections of Rat A2AR TM2 Peptide on Cocaine Self-Administration

Following 14 sessions, rats acquired cocaine (0.25 mg/Kg/infusion) self-administration and displayed less than a 10% variation in the number of infusions received over the last six self-administration sessions. The mean number ± SEM of cocaine infusions per day during the last six self-administration cocaine days was 41 ± 6 cocaine infusions.

Immediately after 14 days of cocaine self-administration (22 h before the test), the rats were microinjected into the nucleus accumbens with vehicle or the A2AR TM2 peptide (0.1 µM/0.5 µL/min/side). On the last day of cocaine self-administration, the rats received a second intra-accumbal injection with vehicle or the TM2 (0.1 µM/0.5 µL/min/side) before injection with the A2AR agonist CGS 21680 (0.1 mg/Kg) or vehicle and the onset of the cocaine session. 

**Figure 1:**
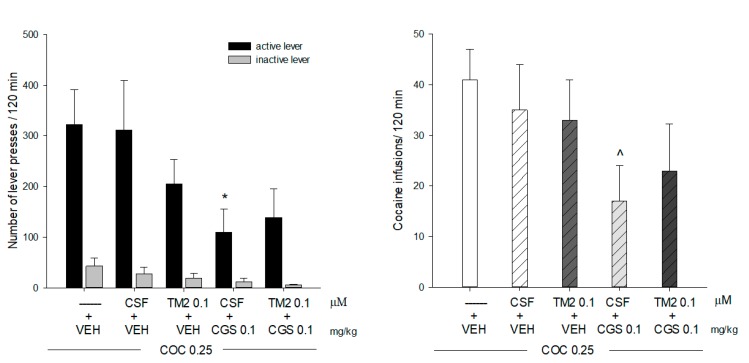
Intra-accumbal effects of the TM2 peptide (0.1 µM/0.5 µL/min/side) or vehicle (CSF; 0.5 µl/min/side) on inhibitory effects of CGS 21680 (CGS; 0.1 mg/Kg; i.p.) under cocaine (COC; 0.25 mg/kg/infusion) self-administration on active and inactive lever presses (**A**) and on cocaine infusions (**B**). Each bar shows the mean±SEM from 7–10 rats/group. * *p* < 0.05 CSF + CGS 0.1 + COC 0.25 vs. VEH (vehicle); ^ *p* < 0.05 CSF + CGS 0.1 + COC 0.25 vs. VEH.

The total cocaine intake after 14 self-administration sessions in groups of rats subjected to various treatment protocols is shown in Table 1. Cocaine intake did not differ in these animals’ groups (F(3.29) = 0.631; *p* = 0.631). (Figure 1). 

CGS 21680 (i.p.) in a dose of 0.1 mg/Kg reduced number of active lever presses (*t* = 2.328, df = 15, *p* = 0.034) and the number of cocaine infusions (*t* = 2.545, df = 15, *p* = 0.022), but had no effect on the inactive lever presses (df = 15, *t* = 1.498, *p* = 0.154) (Figure 1). The intra-accumbal TM2 (0.1 µM/0.5 µL/min/side) changed neither the cocaine (0.25 mg/Kg/infusion) self-administration in terms of active (*t* = 1.361, df = 17, *p* = 0.191) and inactive lever (*t* = 1.208, df = 17, *p* = 0.243) presses or drug infusions (*t* = 0.812, df = 17, *p* = 0.427) nor counteracted the CGS 21680 (0.1 mg/Kg, i.p.) -induced reduction in the number of active (*t* = −0.404, df = 12, *p* = 0.692) or inactive lever presses (*t* = 0.906, df = 12, *p* = 0.382) and cocaine infusions (*t* = −0.569, df = 12, *p* = 0.579) (Figure 1). 

### 2.2. Effects of Intra Accumbal Microinjections of Rat A2AR TM2 Peptide on A2AR-D2R Heteroreceptor Complexes in the Nucleus Accumbens Using In Situ PLA

**Figure 2:**
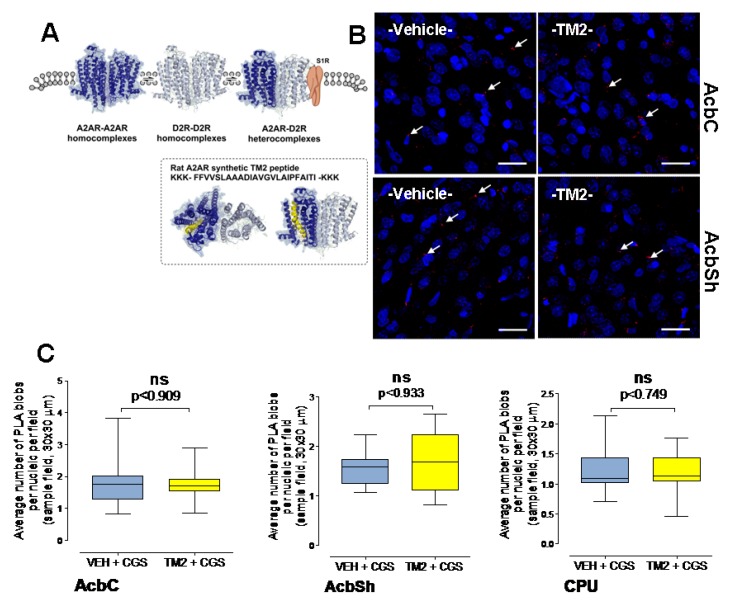
(**A**) A molecular model is shown of the A2AR-D2R heterodimer to illustrate the that the A2AR synthTM2 is not part of the interface (The PDB coordinate of this molecular model was obtained from Borroto-Escuela et al. [8]). (**B**,**C**) Effects of intra-accumbal microinjections of the rat A2AR synthTM2 peptide or vehicle in the presence of CGS 21680 during cocaine self-administration on the density of the PLA positive A2AR-D2R heteroreceptor complexes in nucleus accumbens core (AcbC), nucleus accumbens shell (AcbSh,) and caudate putamen (CPU). (**B**) Representative examples are given of the densities of the red PLA positive A2AR-D2R heteroreceptor complexes after the rat A2AR synthTM2 peptide treatment vs. vehicle in the ventral striatum. (**C**) The microinjection of the A2AR synthTM2 peptide is shown to not alter the density of the PLA positive complexes per nucleic per cell in any of the regions analyzed. Bregma 1.00 mm, Scale bar is 30 μm. Mean ± SEM, number of rats = 5 per group. Student’s *t*-test with Bonferoni correction.

The lack of effects of the A2AR TM2 microinjected into the nucleus accumbens on the densities of the A2AR-D2R complexes are shown in the nucleus accumbens shell, nucleus accumbens core, and caudate putamen regions (Figure 2). Comparing vehicle alone and vehicle/A2A TM2 groups also showed a lack of effect on the A2AR-D2R complexes in any of the regions studied (AcbC, *p* < 0.3922, AcbSh, *p* < 0.0945, Mann–Whitney U test). No significant effects are found upon quantitation of their densities in either region. The density of red PLA clusters is not significantly different (Mann–Whitney U test) with or without treatment with the rat A2AR TM2 peptide. The lack of effects in the PLA are illustrated in the accumbal shell and core (Figure 2). Also, no effects on the A2AR-D2R heteroreceptor complexes were observed in the caudate putamen (vehicle and A2A TM2; give median and semi quartile deviation).

### 2.3. Effects of Intra Accumbal Microinjections of Rat A2AR TM2 Peptide on A2AR-A2AR Homoreceptor Complexes in the Nucleus Accumbens Using In Situ PLA

**Figure 3:**
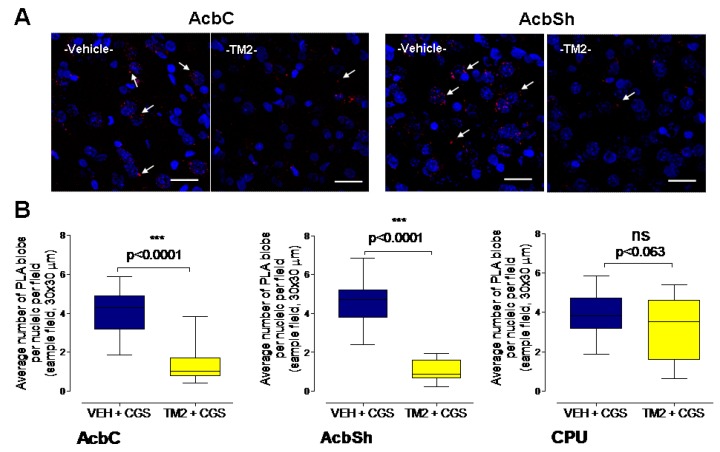
Effects of intra-accumbal microinjections of the rat A2AR synthTM2 peptide or vehicle in the presence of CGS 21680 during cocaine self-administration on the density of the PLA positive A2AR-A2AR homoreceptor complexes in nucleus accumbens core (AcbC), nucleus accumbens shell (AcbSh), and caudate putamen (CPU). (**A**) Representative examples is given for the reduction of the densities of the red PLA positive A2AR-A2AR homoreceptor complexes after the rat A2AR synthTM2 peptide treatment vs. vehicle in the ventral striatum. (**B**) The microinjection of the A2AR synthTM2 peptide is shown to produce a significant reduction in the density of the PLA positive complexes per nucleic per cell in the AcbC and AcbSh. Bregma 1.00 mm, Scale bar is 30 μm. Mean ± SEM, number of rats = 5 per group. Student’s *t*-test with Bonferoni correction.

Highly significant reductions of the A2AR-A2AR homoreceptor complexes were observed by this treatment in the nucleus accumens shell and core (Figure 3). Only a trend for a reduction was found in the dorsal medial region of the caudate putamen (Figure 3). Comparing vehicle alone and vehicle/A2A TM2 groups also demonstrated highly significant disappearance of the A2AR-A2AR homoreceptor complexes in the accumbal regions studied (AcbC, *p* < 0.0001, AcbSh, *p* < 0.0001, Mann–Whitney U test). 

### 2.4. Effects of Intra Accumbal Microinjections of Rat A2AR TM2 Peptide on the Antagonistic A2AR-D2R Interactions in the Ventral Striatum Using [^3^H]-Raclopride/Quinpirole Competition Binding Assay

#### 2.4.1. Vehicle Group Treated with CGS 21680

**Figure 4:**
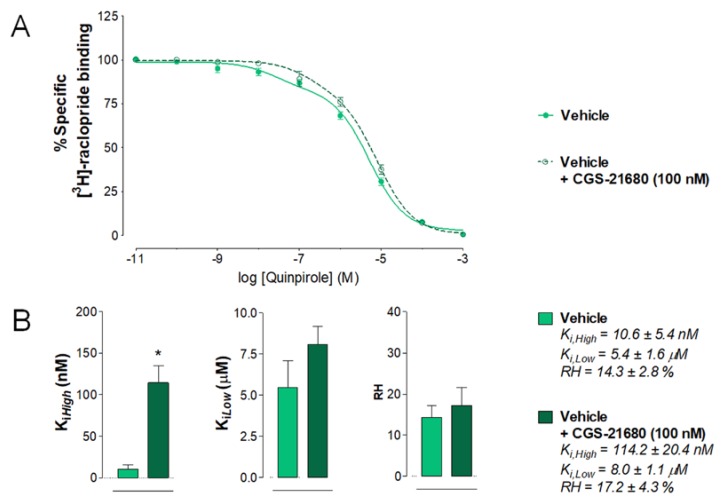
[^3^H]-raclopride/quinpirole competition experiments to determine changes in D2R affinities induced by adenosine A2AR agonist CGS-21680 in the saline-injected (vehicle) rat group (control group). (**A**) Competition experiments involving dopamine D2-likeR antagonist [^3^H]-raclopride binding versus increasing concentrations of quinpirole were performed in ventral striatal membrane preparations from the control group (60 µg/mL) in the presence or absence of the adenosine A2AR agonist CGS 21680 (100 nM) as indicated. Nonspecific binding was defined as the binding in the presence of 100 μM (+)-butaclamol. [^3^H]-raclopride/quinpirole competition curve is based on the values of four rats with each experiment performed in triplicates. The binding values are given in percent of specific binding at the lowest concentration of quinpirole employed. (**B**) Analysis and presentation are given of the A2AR agonist CGS 21680 (100 nM) induced changes in the high-affinity value (K_*i*,*High*_), low-affinity value (K_*i*,*Low*_), and proportion of receptors in the high-affinity state (RH). Means ± SEM are given from four independent experiments performed in triplicates. Statistical analysis was performed by paired Student’s t-test (* *p* < 0.015): the group of rats treated with CGS 21680 is significantly different compared to the group receiving only the saline solution. No significant differences in low-affinity values were observed after CGS-21680 modulation compared to vehicle (*p* = 0.0571 by paired *t*-test). Furthermore, no significant differences in proportion of receptors in the high-affinity state were observed after CGS-21680 modulation compared to vehicle (*p* = 0.5760 paired *t*-test).

Effects of the A2AR TM2 peptide and vehicle on the antagonistic A2AR-D2R interactions were compared in their ex vivo response to the A2AR agonist CGS 21680 (100 nM). As seen in Figure 4A, in the nucleus accumbens membranes of saline-injected rats, a right-shift in the competition curves with quinpirole did develop after adding CGS 21680. The K_*i*,*High*_ value of quinpirole binding to the D2-likeR was significantly increased after A2AR agonist treatment compared to vehicle alone (* *p* = 0.015, paired Student’s *t*-test) (Figure 4B). No significant effects were induced by CGS 21680 on the K_*i*,*Low*_ and RH values (Figure 4B).

#### 2.4.2. A2AR TM2 Groups Treated with CGS 21680

**Figure 5:**
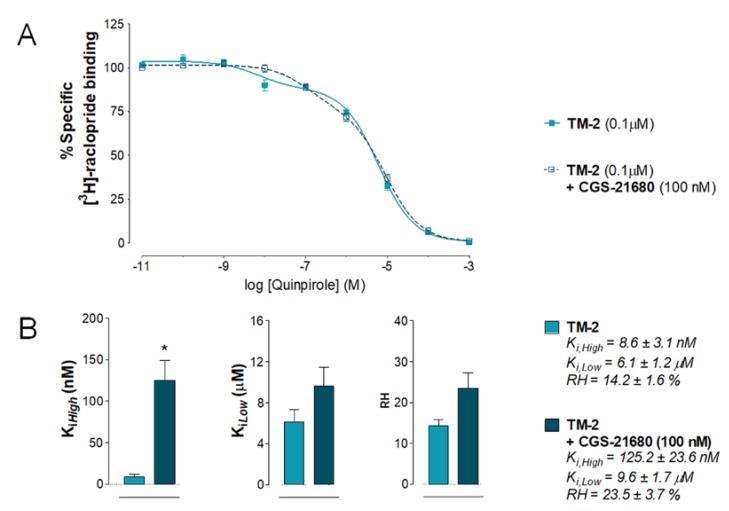
[^3^H]-raclopride/quinpirole competition experiments to determine changes in D2R affinities induced by adenosine A2AR agonist CGS-21680 in the TM2 rat group. (**A**) Competition experiments involving dopamine D2-likeR antagonist [^3^H]-raclopride binding versus increasing concentrations of quinpirole were performed in ventral striatal membrane preparations from the TM2 injected rat group (60 µg/mL) in the presence or absence of the adenosine A2AR agonist CGS-21680 (100 nM) as indicated. Nonspecific binding was defined as the binding in the presence of 100 μM (+)-butaclamol. [^3^H]-raclopride/quinpirole competition curve is based on the values of four rats with each experiment performed in triplicates. The binding values are given in percent of specific binding at the lowest concentration of quinpirole employed. (**B**) Analysis and presentation are given of the A2AR agonist CGS-21680 (100 nM) induced changes in the high-affinity value (K_*i*,*High*_), low-affinity value (K_*i*,*Low*_), and proportion of receptors in the high-affinity state (RH). Means ± SEM are given from four independent experiments performed in triplicates. Statistical analysis was performed by paired Student’s *t*-test (* *p* < 0.019): the group of rats treated with CGS-21680 is significantly different compared to the group receiving only the TM2. No significant differences in low-affinity values were observed in rats receiving TM2 + CGS-21680 compared to rats receiving only TM2 (*p* = 0.0506 by paired *t*-test). Also, no significant differences in proportion of receptors in the high-affinity state were observed in rats receiving TM2 + CGS-21680 compared to rats receiving only TM2 (*p* = 0.0514 paired *t*-test).

In the nucleus accumbens membranes from the TM2-injected rats, the peptide by itself did not alter the affinities of quinpirole binding to the D2-likeR compared to the vehicle-pretreated rats (Figure 4A,B and Figure 5A,B). CGS 21680, as in the vehicle group, induced an increase in the K_*i*,*High*_ value of quinpirole binding to the D2-likeR, which was significantly different compared to rat group treated with TM2 alone (* *p* = 0.019, paired Student’s *t*-test). Under these experimental conditions, TM2 did not block the negative allosteric modulation induced by CGS 21680 on the quinpirole binding to the high-affinity state of the D2-likeR. Furthermore, the K_*i*,*Low*_ and RH values were not affected by the presence of CGS 21680 (Figure 5B).

**Figure 6:**
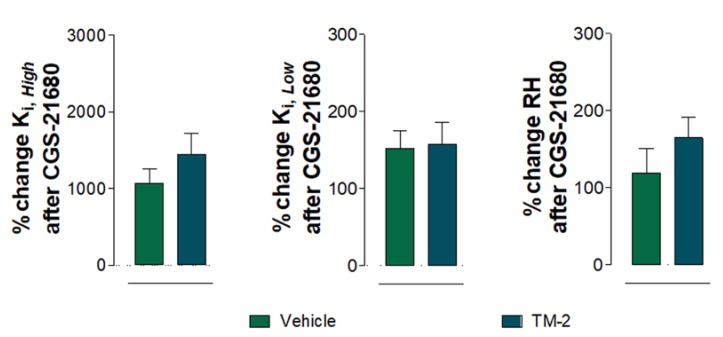
Analysis and presentation of the A2A agonist CGS-21680 (100 nM)-induced percent changes in D2R binding comparing the saline-injected group control (vehicle) and TM2 injected group (given in % of values in the absence of CGS-21680) with regard to the dopamine D2R high-affinity value (K_*i*,*High*_), the low-affinity value (K_*i*,*Low*_), and the proportion of receptor value in the high-affinity state (RH). Means ± SEM are given for four independent experiments performed in triplicate. Statistical analysis was performed by nonparametric Mann-Whitney U test.

The significant percent increase induced by CGS 21680 in the D2R K_*i*,*High*_ values in the vehicle group was not significantly different from the percent increase induced in the A2AR TM2 peptide-treated group. As shown in Figure 6, the percent changes induced by CGS 21680 in the vehicle versus the A2AR TM2-injected animals were not significantly different with regard to changes in the values of D2R _*Ki*, *High*_, D2R _*Ki*, *Low*_, and RH (Mann–Whitney U test).

## 3. Discussion

It is now clear that interactions between adenosine and dopamine take place mainly through the antagonistic allosteric A2AR-D2R receptor–receptor interactions in heteroreceptor complexes of the ventral and dorsal striatum [9,13]. The interface of the A2AR-D2R heterodimer was, in part, characterized [10], and a structural model was constructed with a transmembrane TM4/5 interface [11]. 

The roles of D2R heteroreceptor complexes in cocaine reward and addiction are widely described [14,15,16,17,18,19,20,21,22,23]. Recent preclinical findings coming from our laboratories indicate that A2AR-D2R heteroreceptor complexes present in the nucleus accumbens play an important role in the development of cocaine addiction and may offer a new target for treatment of this severe brain dysfunction [22]. To further support it, cocaine self-administration is enhanced by disruption of the A2AR-D2R heteroreceptor complex following A2AR TM5 peptide administration [12]. The A2AR-D2R heteroreceptor complex is in a dynamic balance with the corresponding homoreceptor complexes and other types of heteroreceptor complexes that share one or more receptor protomers with the A2AR-D2R heteroreceptor complex [24,25].

The major result obtained in the current paper was the demonstration that the A2AR TM2 peptide, which is part of the interface of the A2AR homomer [10,11], produced a disruption of the A2AR homomer complex in the nucleus accumbens shell and core upon its microinjection in this region without altering the A2AR-induced inhibition of cocaine self-administration.

Thus, activation of the A2AR homomer [26] does not appear to play a significant role in the anti-cocaine actions of the A2AR agonist CGS 21680. Another important finding was the demonstration that the A2AR TM2 peptide, when microinjected into the nucleus accumbens, failed to significantly alter the density of the accumbal A2AR-D2R heteroreceptor complexes both in the shell and in the core. These results were supported by findings ex vivo showing that the A2AR agonist CGS 21680 induced significant increases in the D2R K_*i*,*high*_ values in the membranes from the A2AR TM2 peptide-treated rats, which were similar to those found after such treatment in vehicle treated rats. Thus, the antagonistic allosteric A2AR-D2R interactions were unaltered. These results open up the possibility that the disruption of the A2AR-D2R complex induced by the rat A2AR TM5 peptide treatment is specific [12]. However, it is unknown if the A2AR TM5 peptide can also interfere with other A2AR heteroreceptor complexes [27] like A2AR-mGluR5 [28], A2AR-CB1R [29], and A1R-A2AR complexes [5]. Thus, the specificity of the action of A2AR TM5 remains to be demonstrated. The results open up the possibility that the A2AR-D2R heteroreceptor complex may not be built up as an A2A homodimer, forming a complex with a D2R homodimer. Instead it seems possible that the A2AR-D2R remains intact after the disruption of the A2AR-A2AR homomer.

Taken together, the current results provide further evidence that A2AR-D2R heteroreceptor complexes in the nucleus accumbens, through A2AR mediated allosteric inhibition of the D2R protomer, can increase anti-reward in the ventral striatopallidal GABA neurons and inhibit cocaine self-administration. The A2AR homodimer does not appear to be involved in this allosteric mechanism.

## 4. Materials and Methods

### 4.1. Animals

Adult male Sprague–Dawley rats (Charles River, Sulzfeld, Germany) weighing between 260–310 g were used. The animals were housed individually in standard plastic rodent cages in a colony room maintained at 22 ± 2 °C and 45–65% humidity under a 12-h light-dark cycle (lights on at 6:00 a.m.). Rodent food (VRF1 pellets, UK) and water were available ad libitum, except for the period of the initial training sessions when rats were maintained on limited water. 

Following delivery, the animals were allowed to adapt to the environment for at least 1 week before the experiment. The experimental protocols performed in this study were in accordance with the new European Communities Council Directive of September 2010 (2010/63/EU), a revision of the Directive 86/609/EEC. The protocols were approved by the Ethical Committee at the Institute of Pharmacology, Polish Academy of Sciences, Krakow, Poland.

### 4.2. Drugs

Cocaine hydrochloride ((1R, 2R, 3S, 5S)-3-(benzoyloxy)-8-methyl-8-azabicyclo[3.2.1]octane-2-carboxylic acid methyl ester hydrochloride; Toronto Research Chemicals (TRC), Canada) was dissolved in sterile 0.9% NaCl and administered i.v. in a volume of 0.1 mL per infusion. CGS 21680 (Tocris, Bristol, UK, 0.1 mg/Kg) was dissolved in 0.9% NaCl and administrated i.p. 10 min before 2-h self-administration session in a volume of 0.1 mL/Kg. The dose of A2AR agonist was established based on previous behavioral studies [30].

The synthetic peptide used corresponds to the TM2 helix of the rat A2AR (synthetic TM2: KKK-FFVVSLAAADIAVGVLAIPFAITI-KKK). It was purchased from CASLO (Denmark) or VTG (Sweden). At both the *N*- and *C*-terminal juxtamembrane sequence of the rat A2AR synthetic TM2 peptide, the tribasic sequence lysine (KKK) was introduced, to ensure incorporation into the plasma membrane of the cell. The synthetic TM2 peptide (0.1 μM) was diluted in the vehicle solution (an artificial cerebrospinal fluid Krebs-Ringer composed with (in mM) NaCl 120, KCl 2, MgCl_2_ 1.8, CaCl_2_ 1.2, Na_2_SO_4_ 0.5, NaHCO_3_ 20, KH_2_PO_4_ 0.5, D-glucose 6.8, pH 7.4) and administered into nucleus accumbens twice (22 h and 20 min) before the last cocaine self-administration session in a volume 0.5 μL infused during 1 min bilaterally. The amount given each time was 0.05 pmol, leading to a total amount of 0.1 pmol per side. The TM2 or vehicle was injected into the nucleus accumbens at a constant flow rate (0.5 μL/min) per side.

### 4.3. Surgery

After eighteen hours, water-deprived rats were trained to press a lever for 2 h daily in standard operant chambers (Med-Associates, Fairfax, VT, USA) under a fixed ratio 5 (FR1-5) schedule of water reinforcement. Two days following lever press training and free access to food and water, animals were anesthetized with ketamine HCl (75 mg/Kg, i.m.; Biowet, Puławy, Poland) and xylazine (5 mg/Kg, i.m.; Biowet, Puławy, Poland) and chronically implanted with a silastic catheter in the external jugular vein, as described previously [31]. Immediately after the catheter implantation, rats were stereotaxically implanted with stainless steel bilateral guide cannuala (22-gauge, 10 mm long; Plastic One, Roanoke, VA, USA). Guide cannula was implanted into the nucleus accumbens shell at the following coordinates from the Bregma: (anteroposterior (AP) = 1.7 mm; mediolateral (ML) = ± 0.75 mm; and dorsoventral (DV) = −6 mm) according to the atlas of Paxinos and Watson (1998). The guide cannuala was affixed to the skull with two miniature stainless steel screws (Agnatho’s, Sweden) and dental acrylic cement. During the first 3 days after catheter and cannula implantation, once daily meloxicam (Metacam, Boehringer IIngelheim; 5 mg/Kg, *s.c*.) was used to reduce post-operative pain in rats. Rats were allowed 9 days to recover from surgery before the start of the experiments. Catheters were flushed daily with 0.2 mL of saline solution containing heparin (100 U/mL, Biochemie GmbH, Kundl, Austria) and 0.1 mL of a cephazolin solution (100 mg/mL Biochemie GmbH, Kundl, Austria) to prevent catheter non-patency. No problems with catheter patency were reported in the tested rats.

On experimental test day, internal cannualae (28-gauge, 12-mm length, Plastics One, USA) were inserted into the guide cannulae (internal cannulae extending 2 mm beyond the end of guide cannula) after obturator removal. The microinjection volume of 0.5 μL was delivered bilaterally over 1 min by microinjection pump (CMA/Microdialysis, Dalvägen, Sweden).

### 4.4. Cocaine Self-Administration

After the recovery period, all animals began lever pressing for cocaine reinforcement during 2-h daily sessions performed 6 days per week. The procedure was carried out as described previously [30]. Cocaine self-administration procedure was conducted under FR5 schedule of reinstatement for 15-daily 2-h sessions. Following each injection, there was a 20-s time-out period during which responding was recorded, but had no programmed consequences. Presses on the ‘inactive’ lever were recorded, but not reinforced. After the 7 days of acquisition, rats were used to complete a 0.5 mg/Kg/infusion of cocaine followed by a 0.25 mg/Kg/infusion of cocaine. Following stabilization of responding rates with cocaine (0.25 mg/Kg/infusion) self-administration, the animals were divided into separate groups (*N* = 7–10) to undergo test procedures. Tests were performed on the last two days of self-administration session. Following the last 2-h cocaine self-administration session, the animals were sacrificed by decapitation (for radioligand binding assays) or they were injected with an overdose of pentobarbital and perfused intracardially with saline followed by perfusion with 4% paraformaldehyde solution to produce exsanguination (for in situ PLA experiments).

### 4.5. In Situ Proximity Ligation Assay (In Situ PLA)

The analysis of A2ARTM2 synthetic peptide’s effects (synthTM2 peptide) on the A2AR-A2AR homoreceptor and A2AR-D2R heteroreceptor complex densities after cocaine self-administration was performed using the in situ PLA method as described previously [24,32,33]. After the last cocaine self-administration session, rats were immediately injected with pentobarbital (133.3 mg/kg, i.p.; Biowet, Puławy, Poland) and perfused intracardially with saline followed by perfusion with 4% paraformaldehyde solution (VWR, Gdańsk, Poland). Each brain was quickly removed, chilled in ice-cold saline, and immersed in the same fixative for 12 h. The brain was left at 4–8 °C in 10% *w*/*v* sucrose up to 7 days followed by 30% *w*/*v* sucrose for two weeks. Each brain was dissected out into the nucleus accumbens and caudate putamen, while samples were immediately frozen on dry ice and stored at −80 °C. Free-floating formalin fixed brain sections (30 μm-thick, cut using a cryostat) at Bregma level (1.0 mm) from rats after cocaine-self administration were employed using the following primary antibodies: rabbit monoclonal anti-A2AR (AB1559F, 1:250; Millipore, Sweden), mouse monoclonal anti-A2AR (05-717, 1:500, Millipore, Sweden), and the mouse monoclonal anti-D2R (MABN53, 1:600, Millipore, Sweden). Control experiments employed only one primary antibody or cells transfected with cDNAs encoding only one type of receptor. The PLA signal was visualized and quantified by using a Leica TCS-SL SP5 confocal microscope (Leica, Allendale, NJ, USA) and the Duolink Image Tool software. Briefly, fixed free-floating rat brain sections (storage at −20 °C in Hoffman solution) were washed four times with PBS and quenched with 10 mM glycine buffer, for 20 min at room temperature. Then, after three PBS washes, incubation took place with a permeabilization buffer (10% fetal bovine serum (FBS) and 0.5% Triton X-100 or Tween-20 in Tris buffer saline (TBS), pH 7.4) for 30 min at room temperature. Again, the sections were washed twice, 5 min each, with PBS at room temperature and incubated with the blocking buffer (0.2% BSA in PBS) for 30 min at room temperature. The brain sections were then incubated with the primary antibodies diluted in a suitable concentration in the blocking solution for 1–2 h at 37 °C or at 4 °C overnight. The day after, the sections were washed twice, and the proximity probe mixture (minus and plus probes, for details see: Duolink instructions) was applied to the sample and incubated for 1 h at 37 °C in a humidity chamber. The unbound proximity probes were removed by washing the slides twice, 5 min each time, with blocking solution at room temperature under gentle agitation. The sections were incubated with the hybridization-ligation solution (BSA (250 g/mL), T4 DNA ligase (final concentration of 0.05 U/µL), Tween-20 (0.05%), NaCl 250 mM, ATP 1 mM, and the circularization or connector oligonucleotides (125–250 nM)) and incubated in a humidity chamber at 37 °C for 30 min. The excess of connector oligonucleotides was removed by washing twice, for 5 min each, with the washing buffer A (Sigma-Aldrich, Duolink Buffer A (8.8 g NaCl, 1.2 g Tris Base, 0.5 mL Tween-20) dissolved in 800 mL high purity water, pH to 7.4) at room temperature under gentle agitation and the rolling circle amplification buffer was added to the sections and incubated in a humidity chamber for 100 min at 37 °C. Then, the sections were incubated with the detection solution through hybridization (fluorescent oligonucleotide probes) in a humidity chamber at 37 °C for 30 min. In a last step, the sections were washed twice in the dark, for 10 min each, with the washing buffer B (Sigma-Aldrich, Duolink Buffer B (5.84 g NaCl, 4.24 g Tris Base, 26.0 g Tris-HCl), dissolved in 500 mL high purity water, pH 7.5) at room temperature under gentle agitation. The free-floating sections were put on a microscope slide and a drop of appropriate mounting medium containing DAPI giving a blue staining of the nuclei (e.g., VectaShield or Dako) was applied. The cover slip was placed on the section and sealed with nail polish. The sections were protected against light and stored for several days at −20 °C before confocal microscope analysis.

### 4.6. Biochemical Binding Experiments

#### 4.6.1. Membrane Preparation

Frozen tissue was homogenized in ice-cold preparation buffer using a sonicator (Soniprep 150). The buffer contained 50 mM Tris-HCl, pH 7.4, 7 mM MgCl_2_, 1 mM EDTA, a cocktail of protease inhibitors (Roche Diagnostics, Mannheim, Germany), and 0.3 IU/mL adenosine deaminase (EC 3.5.4.4, Sigma-Aldrich). The membranes were precipitated by centrifugation at 4 °C for 40 min at 40,000× *g* (Thermo scientific, Sorvall Lynx 6000, Stockholm, Sweden) and washed through re-homogenization in the same buffer once more. The protein concentration was determined for the membrane homogenates by means of BCA Protein Assay (Pierce, Sweden) using as a standard bovine serum albumin. Pelleted membranes were resuspended to a concentration of 0.15 mg/mL, immediately used or stored at −80 °C until required.

#### 4.6.2. [^3^H]-Raclopride Competition Binding Experiments

[^3^H]-raclopride binding was displaced by quinpirole to determine the proportion of receptors in the high-affinity state (RH), the high-affinity (Ki, High), and low-affinity (Ki, Low) values. Nucleus accumbens membrane preparations (60 μg protein/mL) were incubated with increasing concentrations of quinpirole (0.01 nM to 1 mM) and 2 nM [^3^H]-raclopride (75 Ci/mmol, Novandi Chemistry AB, Sweden) in 250 μL of incubation buffer (50 mM Tris-HCl, 100 mM NaCl, 7 mM MgCl2, 1 mM EDTA, 0.05% BSA, 1 mM DTT) and 0.3 IU/mL adenosine deaminase (EC 3.5.4.4, Sigma-Aldrich) for 90 min at 30 °C in the presence or absence of 100 nM of the A2AR agonist CGS 21680. Nonspecific binding was defined by radioligand binding in the presence of 100 μM (+)-butaclamol (Sigma-Aldrich, Sweden). The incubation was terminated by rapid filtration Whatman GF/B filters (Millipore Corp, Sweden) using a MultiScreenTM Vacuum Manifold 96-well followed by five washes (250 μL per wash) with ice-cold washing buffer (50 mM Tris-HCl pH 7.4). The filters were dried, 5 mL of scintillation cocktail was added, and the amount of bound ligand was determined after 12 h by liquid scintillation spectrometry.

### 4.7. Statistical Analysis

Data were analyzed using *Statistica 13* (Statistica, USA). All the data are shown as means ± SEM. In behavioral experiments, the number of total cocaine infusions was analyzed using a one-way analysis of variance (ANOVA). The number of active and inactive lever presses as well as cocaine infusions for different groups were analyzed with an unpaired Student’s *t* test.

In neurochemical experiments, the number of rats in each experimental condition is indicated in figure legends. Data from competition experiments were analyzed by nonlinear regression analysis using GraphPad Prism 5.0 (GraphPad Software Inc., San Diego, CA, USA). The absolute values and the percent changes induced by A2AR agonist CGS 21680 in the D2R high-affinity binding, low-affinity binding, and proportion of receptors in the high-affinity state were evaluated with paired Student’s *t*-test and nonparametric Mann–Whitney U test respectively. Data from in situ PLA experiments showing cluster density (clusters per nucleic per sampled field) were analyzed using a one-way ANOVA followed by post-hoc Tukey’s test. The *p* value 0.05 and lower was considered significant.

## Figures and Tables

**Table 1:** The total cocaine intake after 14 cocaine self-administration sessions in rats.

Treatment	No. Rats	Total Cocaine Intake (mg/rat) [Mean ± S.E.M]
Vehicle (intra accumbal) + Vehicle (i.p.) + cocaine (i.v.)	10	173 ± 26
TM2 (intra accumbal) + Vehicle (i.p.) + cocaine (i.v.)	9	224 ± 49
Vehicle (intra accumbal) + CGS 21680 (i.p.) + cocaine (i.v.)	7	213 ± 24
TM2 (intra accumbal) + CGS 21680 (i.p.) + cocaine (i.v.)	7	174 ± 16

## Abbreviations

A2AR	Adenosine A2A receptor subtype
D2R	Dopamine D2 receptor subtype
GPCR	G protein-coupled receptor
PLA	Proximity ligation assay
TM	Transmembrane
